# Selections and Abstracts

**Published:** 1901-01

**Authors:** 


### SELECTIONS AND ABSTRACTS.

### REMINISCENCES OF A PHYSIOLOGIST.*

LADIES AND GENTLEMEN:—When my old friend, whom I am proud to call my old pupil, Dr. Sewall, asked me soon after my arrival in Denver whether I would be willing to say a few words to you, I, who have always suffered all my life from the inability to say "no," at once said "yes." But I must confess that I am not "in good form" to make you a formal address. I am "on the tramp" over your vast continent and making east as fast as ever I can. All that I can offer you this morning is some very rambling, gossiping chat.

Dr. Sewall has suggested that I should say a word or two about early days. It was in the year 1854 when I began my medical studies; but I had the year before attended a course of lectures on physiology, breaking into my ordinary studies in order to do that, and my teacher was a man by the name of William Sharpey,—a very great man, but a man whose name, perhaps, will not occupy the place as that of a great physiologist, which it really deserves. Those of you who have studied the structure of bone will remember his name under the title "Sharpey's Fibres." Indeed, he was one of the first to give an accurate description of the true structure of bone. He was at that time the only pure physiologist in England. He had acquired a very great reputation many years before, in the early '30's—I forget the exact year, '32 or '33, I think—by discovering cilia. Before that time all the movements which are produced by cilia were a mystery. And almost in the same year Valentin, who was professor of Physiology at Bern, and Sharpey, who was at that time teaching anatomy at Edinburgh, discovered cilia. It is worthy of note that both of them discovered these cilia, not with the compound microscope, but with the simple lens. That gave them at that time far more trustworthy results than the indis-

*An Address delivered by Sir Michael Foster, K.C.B., M.D., F.R.S., to the Students of the Denver College of Medicine, September 13, 1900.

tinct or untrustworthy images which were gained by the compound microscope. Sharpey may perhaps be known to you also as the editor of a book, which for years and years has been and still is a standard work on anatomy in England, Quain's Anatomy, which deals not only with topographical anatomy, but also with minute structure, with what we now call histology; and Sharpey was the first man to teach histology in a thoroughly systematic manner in England. There had been in years before, in the '40's, a man who did most remarkable pieces of work in histology, one on muscle and the other on kidney, William Bowman, works which remain to the present day classical works on both those tissues; but he, already in 1850, was a practical surgeon, had a large practice in ophthalmic surgery, and had to abandon the active pursuit of physiology. Sharpey was the only man at that time who devoted his whole life to physiology. In all the other schools physiology was taught by practicing physicians and surgeons. He had had a long experience, a long training; and when I tell you that Sharpey was the pupil of Rudolphi, the greatest physiologist in Berlin at the end of the last century and the beginning of this, the master of Johannes Mueller, who really began modern physiology, and whose pupils were Helmholz, Ludwig, du Bois Reymond and others, you will recognize that his name carries one a long way back.

Now Sharpey was, at the time I am speaking of, the greatest physiologist in England, the only person who devoted his whole time to science; and yet even he taught physiology entirely by lectures. He had no physiological laboratory. He had no physiological apparatus whatever. All he did in the way of practical teaching at that time was to show us under the microscope preparations of the various tissues. There was no attempt whatever at any practical teaching of physiology. I remember very well that when he was lecturing on blood-pressure, and was describing to us the then new results of Ludwig, endeavoring to explain to us the blood-pressure curve, all he had to help him was his cylinder hat, which he put upon the lecture table before him and with his finger traced upon the hat the course of the curve. That was the way that physiology was taught by Sharpey in England in the year 1854. And yet Sharpey taught it as nobody else taught it. Nobody else in England then was teaching physiology as Sharpey taught it, and, as I tell you, he used his hat, and a very old hat it was, as a kymographion, for blood-pressure.

I very well remember going to him one day after his lecture, in which he had been speaking of the functions of the liver (by that time he had recognized that I had a special interest in physiology), and he said to me, "Well," he said, "I didn't like to say anything about it in my lecture, but Claude Bernard in Paris has just sent me a paper which he has read before the Academy of Sciences at Paris, and in that paper he has proved that there is present in the liver a substance resembling starch, which is easily converted into sugar." I said to him, "Good gracious, that is something quite new, isn't it?" And he said, "Yes, and probably next year in my lectures I shall be able to tell the rest of the students about that, but I thought you would like to hear about it." That was Claude Bernard's discovery of glycogen.

Well, after I had taken my qualifications and become "a healer of the sick," I went into practice for about six years. Some of my patients liked me. I myself wondered very much at it. I am very thankful that I did no more mischief than I did during those six years of practice. After those six years I was called back to London, and by that time there had been some change. At University College, one of the medical schools connected with the hospitals in London, and one which was certainly at that time in advance of many of the other medical schools, they had, with the help of Sharpey, who was then becoming an old man, established a lectureship on practical physiology, not a full professorship, but a mere lectureship, a subordinate position which was held by Dr. George Harley, who did some very good work in his time. That was the first attempt in London to teach physiology in a practical manner. Just about the same time Dr. William Rutherford, whose name is probably familiar to some of you, especially for his work on the vagus nerve, began, under Professor Hughes Bennett, to teach practical physiology in Edinburgh. I was called to succeed Dr. G. Harley as teacher of practical physiology. That was in the early '60's, in about '64 or '65. But what could be done then was very little. I had a very small room. I had a few microscopes. But I began to carry out the instruction in a more systematic manner than had been done before. For instance, I made the men prepare the tissues for themselves. That was a new thing then in histology. And I also made them do for themselves simple experiments on muscle and nerve and other tissues on live animals. That, I may say, was the beginning of the teaching of practical physiology in England, which has become what it is in the present year in the years that have elapsed between then and now.

Now, I came up to London from the country. I had been a country doctor, a doctor in a small country town where I used to work at my physiology whenever my patients would leave me alone. I came up to London and began this course of lectures on practical physiology. I appointed a time to see the few who wished to enter upon my course. That course was absolutely voluntary. In England, as here, I suppose, certain courses are compulsory. Anatomy was then compulsory, the ordinary lectures on physiology were compulsory, and other lectures were compulsory; but these lectures on physiology were absolutely voluntary, and only the better students were willing to give up the time needed to get a more thorough grasp of physiology. Well, I appointed a time to see the few who wished to spend some time in this new study, this study of luxury, and there came to me a boy, nothing more than a boy, at least he looked a boy, who said, "I am very sorry, sir; I should like to take your course if I could, but you see my parents are not very well off, and I get my board and lodging by living with a doctor close by." Doctors in England then, as indeed they do very largely now, dispensed their own medicines. I mean, when they saw a patient they sent in afterwards the medicines required. In those times medicines were not so compendious as they are now; the doctor could not take the whole pharmacopœia about in a little case. He, either with his own hand or by help of an assistant, had to do a good deal in the way of preparation of medicines, making infusions, decoctions, rolling pills and making up mixtures and draughts, doing all the things which went under the general name of dispensing. The lad I am speaking of said to me: "I have, in return for my board, to dispense all the doctor's medicines, and that dispensing takes me always from 2 to 5; now your lectures begin at 4. I cannot come for the first hour. You go on to 6. May I come in for the second hour? I will work hard and will try to make up the lost time." I said, "Certainly, certainly." So he came in, came in regularly late. The other boys rather laughed at his coming in late. He came in regularly at 5 o'clock, and he worked with such purpose that in the examination which I had at the end of the course I awarded him the prize. Well, his name was Henry Newell Martin, and I was so much struck with him that I asked him to assist me in my course, and he become my demonstrator. After we had been at University College together I think two or three years, Martin carrying on his studies and at the same time helping me, he came one day to me in great trouble because he could not make up his mind. He obtained what they called a scholarship at Christ College at Cambridge, and he could not make up his mind to accept it and go there. He said he didn't want to leave me. But I was able to tell him what nobody else knew at the time, that in the October in which his scholarship would take him to Cambridge I was going to Cambridge, too, having been invited to be a lecturer on physiology there. So we both went to Cambridge at the same time, and he became at Cambridge at once my demonstrator, as he had been in London. And after a career of considerable brilliancy of some years at Cambridge there came to him an invitation to Johns Hopkins University at Baltimore. So, if I have done nothing more, at all events I sent Henry Newell Martin to America. (Applause.)

Well, now, I cannot talk about medicine generally, but perhaps I may say a few words about the examinations which were held in those days for entrance into the profession. Examinations form a subject of contention. There is a good deal to be said on one side and on the other side. I think there is no doubt that examinations are necessary, but I think also that in England, at all events, and possibly here, examination are often abused. Examinations, instead of being made, as they ought to be made, simply an inducement to proper study, become an inducement to that which is sometimes called study, but which is not study at all, which in England we call simply "cramming up," by which a lad often passes the examination without knowing anything really of the subject on which he is examined. I remember talking to a man whose duty it was to prepare and fit—I hardly know whether I should say fit or unfit—people for examinations. I think it was the examination for entrance into the army in England. One day the list came out and the first four men on the list were all his pupils, and I congratulated him. I said, "You have done very well this time; all these four men are your men." "Oh," he said, "I am not proud of that. Those were clever boys and they knew their subjects. What I am proud of is number eighteen; he has passed, but he knows absolutely nothing whatever about it." (Laughter.)

Now, as I said, I went down into the country authorized to take care of the sick, and my authorization took place in this wise. Before I had entered the professon I passed a preliminary examination to show that I knew a little Latin, a little Greek, a little arithmetic, and a little geography, or something of that kind. Then I had to attend regular courses of study for three years. At the close of these I "went up," as we say, to the Royal College of Surgeons. The diploma is issued by that body, which is simply an examining body. It doesn't teach at all. Diplomas are issued by it, and nearly every doctor in England becomes a member of the College of Surgeons. Nowadays he usually becomes at the same time a member of the College of Physicians; but a change has taken place since my time. At that time many men had as their sole qualification the diploma of the Royal College of Surgeons. I proposed to take my degree of doctor of medicine in the University of London; but like many others, I desired to have as well this diploma of the College of Surgeons, which was the usual qualification to practice medicine. The examination for that diploma I underwent at the end of my studies. It took place in the evening. I think we went there about 7 o'clock in the evening. At about 8 o'clock a bell rang and four of the candidates were sent in to be examined.

The examination to some of us, I am sure, was anything but easy. I have a distinct recollection of a man, one of my fellow candidates, who had already been up several times for the examination before. As we were waiting for the examination we talked, of course, about the possible questions and the proper answers, and the man in question asked us: "Now, what answer would you give to this question: 'If a man got between the buffers of an engine what would be the injury which would result?'" Well, we all talked about rupture of the liver and a variety of things. "Oh, no," he said, "that is not the answer. What you have got to say is, 'there will be an ecchymosis.'" I believe he was rejected on that evening, as he had been on previous evenings.

The examination was conducted in this way. We four were ushered into a room, and there sat at four tables eight men, two men at each table, who seemed to us to glare at us as we were ushered before them. Then each of us sat at a table, and I found I was being examined in anatomy by a man who was an eminent surgeon. He asked me questions in anatomy and I went on struggling with my answers. He went on giving me as many questions as he could for a quarter of an hour, and then the bell rang, whereupon it was like a certain game the children play, we all changed tables as rapidly as we could. I got at another table and I found I was being examined in physiology; that was not by a physiologist, but by a physician. The first question he asked me was about the action of the muscles of the larynx. Well, as you know, there are many answers to questions about the action of the muscles of the larynx, and I entangled him into a discussion about the action of these muscles and we went at it for a quarter of an hour, and then the bell rang again. My examiner said, "Good gracious, I have hardly asked you anything." And then I had a quarter of an hour at surgery and afterwards I had a quarter of an hour at pathology, and then we all went out; and that was the examination. We waited for about half an hour or so and then certain names were called, and these had joy on their faces, but the poor fellows whose names were not called went away sadly, for they had not been accepted. We who had been called had an address by the president of the College of Surgeons, and then each had a piece of paper handed to him. On the strength of the result of that one hour I was given leave to work my will on any poor person who had the misfortune to consult me. (Laughter.)

Well, things have changed a good deal since then, and changed for the better. As I said, the subject of the examination is one thing which requires continual care lest it should defeat its own end.

Now about my other studies. A year or two after I came up to London I was asked to deliver a course of lectures at the Royal Institution, and in talking with my dear old master Sharpey about what I should select for a course of lectures, I cannot remember whether I suggested it to him or whether he suggested it to me, but anyhow we determined that I should give a course of lectures on embryology. Well, I knew practically nothing about embryology, and indeed very little was known in England; certainly very little knowledge was required of the student. There was a man who had worked at it, Allen Thompson, and a great deal of work had been done, of course, in Germany, for Koelliker was hard at work at his embryological researches. He had by that time done a great deal, and was about to issue his well-known work on the subject; but in England nobody was working on the subject save Huxley. Huxley had for purely morphological purposes gone very fully into the development of the chick in the egg, and he had written a few papers about it, but beyond him nobody, so far as I know, was then working; at all events at vertebrate embryology. I had learned nothing except what dear old Sharpey had told me in the course in physiology, and I began of course to set to work to learn a little about the subject before I began to lecture on it.

Now what I am about to say just illustrates the progress which has been made since that time. This was in the early '60's. I remember Sharpey telling me (he had worked with Allen Thompson) that the way Allen Thompson and he obtained a new view of the structure of the embryo was to cut the embryo in half with a razor and look at the two cut surfaces with a strong glass. They made no sections whatever, and Sharpey said to me when I began, "You know Huxley tells me that Koelliker has a capital method of getting sections of the embryo. He spreads the blastoderm flat on a glass plate, and then taking hold of each end of the blade of a razor he brings it down again and again on to the blastoderm until he thus cuts the embryo up into a number of slices, and then he takes out the thinnest ones for examination under the microscope.

Well, I used the razor to prepare my sections, as that was the only tool for that purpose we then had. But I used also the method of embedding. I shall never forget the delight of dear old Sharpey when I showed him some sections (horribly rough sections they were. I shouldn't like to have them exhibited now, but I was proud of them at that time) of the embryo hardened and embedded. He was absolutely delighted. "Oh," he says, "I wish Allen Thompson could see those." I believe I was one of the very first in England to use the method of embedding for microscopical sections. At all events, I was one of the first. It was just about that time that it came into use in England. As you know, what is now the technique of histological examination, how almost absolutely complete and perfect it is; all that has grown up since I put my hand to the work.

Now I think I will turn to a somewhat different topic. If I may, I should like to say a word or two about vivisection, because that is a subject of very great interest to the medical profession. I should just like to tell you what our experience is in England. I think it was in the late '60's or the beginning of the '70's there was a movement for putting the performance of experiments on living animals under state regulation. By an experiment was meant something done with a view to get the truth, not for any other purpose. The object of the operation must be simply to get at the truth, if so, it was called an experiment on living animals, and it was proposed to put that under state regulation. There was a commission which sat on the subject and reported upon it, and then an act of Parliament was passed as the result of the report of that commission. And I think I may say there was a very general opinion among those who were unbiased that the report of the commission went beyond the evidence, and that the action of the Legislature went beyond the report. I do not believe myself that there was any necessity whatever for such legislation. However, such legislation was carried out.

Now that legislation consists of two parts, one of which does not put any great restraint upon inquiry, but the other does. One part is that every one who performs experiments on living animals for the purpose of ascertaining the truth must possess a license.

Well, there is no great hardship in that. The application for the license has to be signed by certain persons of repute, and there is no difficulty at all in any person who is capable of carrying out proper experiments on living animals obtaining a license. If there was simply the obtaining of a license, I don't think that act would have had any prejudicial influence at all. But there were other parts to the act. One was that the place in which the experiments were to be performed must be recognized and licensed as a place for performing experiments on living animals. Now that had and still has a very prejudicial effect. The object of that was that the person who was performing the experiments might be subject to supervision by an inspector; an inspector could know where the experiments were being carried on and come down at any time to see whether the experiments were being carried on according to law. Although it had no prejudicial influence upon professional physiologists, it had this, and I think a very bad effect, of proving a great obstacle to men in practice performing experiments with a view of carrying out their research. Before that a surgeon or a physician who was undertaking a reseach could perform an experiment in his own house, but after that act no one could perform an experiment in his own house unless he had his own house licensed as a place for the performance of experiments, and the licensing of his house made public by a Return to Parliament, and this practically cut off a very large number of active practicing physicians and surgeons from carrying out research. I know that Lister himself, who did a great deal of his work in his own house, and of the immense value of that work you are well aware, has said that it would have been impossible for him to have carried out his researches if he had had to have carried them out after that act had become the law of the land.

Then there is another thing which is a very great obstacle, and that is that the license will not cover all experiments. It will only cover ordinary experiments. It will, for instance, not cover experiment on cats or dogs or mules or monkeys or horses, and I think some other animals. If you want to perform an experiment on one of those animals, although you have a license to experiment on living animals, you must get, in addition, a certificate for the special experiment which you want to carry out. Now, that license has to come through the government, has to come through what we call the Home Office. I don't know how you do things in America, but in England whenever anything gets into a public office there it remains for a very considerable time before it gets out; and by the very force of circumstances, not by reason of any ill will, since the Home Office, now at any rate, recognizes that our objects are good and gives us every assistance it can to carry out the work when it is clear there is intent to carry it out as the act was intended to be carried out, but a considerable delay occurs. The certificate system acts in the following way. You have undertaken a research, your license carries you on along a certain line, and then you want to make a special experiment outside that line. For this you must have a special certificate, and you must suspend the research until you get your special certificate. All of you know that it is impossible to wait and put a research aside without becoming interested in some other subject. I know that I have more than once commenced work in certain directions, and then have had to apply for a special certificate, and I have put my work aside, and by the time the special certificate reached me I had gone off into some other line and my mind was wholly occupied with that and I never went back to the research for which I had obtained certificate. The certificate was wholly useless.

Now that has occurred to me, and that has occurred to everybody else, and I need hardly say in a research in science it is of the utmost importance that you should be able to take up any subject which comes to you and follow it out at once. You must strike while the iron is hot. You can't do that under the present condition of things in England, that has, I am very sure, produced a very prejudicial effect upon the progress of physiology in England. Of course, there is the other side of the question. I suppose you don't experience the sort of thing here, but in the rest of the world we are stimulated by difficulties; and I think there can be no doubt that the fact we have to fight against difficulties in the progress of physiological inquiry occasioned by the act has really stirred us up to more active and more earnest inquiry than if the act had not passed; but if the question comes before you, don't you be too much influenced by the beneficial effects of difficulties. (Laughter.) Resist, as I have said to every one in America, resist interference with your inquries. I heard the Lord Chief Justice of England, when this matter was being discussed in the House of Lords, I heard him say: "You must remember, my Lords, that this is essentially a penal act." I, a professed physiologist, sat in the gallery of the House of Lords, and heard the highest legal authority state that the Legislature were about to treat me as a felon. They were about to bring into action what he called a penal act. Don't you allow yourselves for a minute to get crippled by all these special certificates.

Indeed, no legislation at all is necessary. I speak not for myself, I speak for all my brethren. We are not cruel. We never cause pain if we can possibly help it, and the whole progress of physiology is dependent upon the experiments upon living animals. The other people may say what they like, but if you read the whole story of physiology you will see that every step has been based upon an experiment on living animals. I have just come from delivering a course of lectures in San Francisco on the history of physiology. I always knew that our science was based upon experiment, but I never knew it as I know it now when I have had to read carefully the old authorities and trace out all their work. Every one who has made an advance has made it by experiment on living animals. And in the old time, for instance in the seventeenth century, in the '60's or '70's of the seventeenth century, just following Harvey, there was a man, Richard Lower, who did very great work on the circulation, on respiration and other parts of physiology, and so far as I can make out from reading his works he must, in the quiet retreat of Oxford, have performed just as many experiments, indeed, I believe on the whole he performed in the same time more experiments than Claude Bernard; and Harvey himself, in his great work on the heart, says not only once, not twice, but several times, that he came to the truth first of all by observing certain facts in the animals and then testing those facts by repeated experiments on living animals. And it is a duty of the whole medical profession to see that the physiologist is not hampered in his important inquiries by any misdirected legislation. (Applause.)

Now I have only just one word more that I should like to say, and it is about the relation of physiology to medicine, and I can speak not as a pure physiologist, but one who has known all the joys and all the sorrows of practice in the country. I have two things to say, and the first is that physiology must be cultivated as a science, as a pure science perfectly irrespective of the medical profession. It is an abstract science of the same value as chemistry or morphology, or any other of the fundamental sciences. It is one of the fundamental sciences and it must be cultivated as such, and it must be ranked as such, and it must hold its position in any university curriculum as an independent science. But, on the other hand, it has direct relation to medicine. In respect to this I desire to say, and I speak now, as you see, from very many years of experience, that I think that it is not only a great hardship but a great mistake to think that every one who is going into the medical profession should be an accomplished physiologist, knowing all, may I call them the "tips" of recent discoveries. All that is needed is that he should have a fundamental knowledge of the subject, a true, solid grasp of the fundamental principles. This may be taught him, and taught him properly, in a very limited time; and if he knows those principles than he can carry this physiology into his pathology, and he can carry both of them to the bedside. And in this relation physiology is a knife which cuts both ways, and I don't know which edge is the sharpest. If you have a firm grasp of the fundamental principles of physiology and of pathology you will be able to thoroughly understand any new view which may be brought before you. And here comes the double edge—You may recognize the new view as something which is based upon the true principles of physiology and pathology. Or, and this, I think, happens quite as often as the other, you may see that the new view is wholly antagonistic to the true fundamental principles of physiology and pathology; and seeing that will help you to clear your mind and prevent your being led away by enticing but erroneous and valueless theories. While I would urge as a physiologist that physiology should always be one of the important introductory studies to the medical profession, there ought to be the greatest care that only, if I may say so, the real juice of the grape of physiology is given to the medical student, and that his mind is not burdened by all the passing fancies of the day, which naturally occupy a great deal of the time of the professional physiologist, but for the most part vanish when they are brought into contact with practical life. (Great applause.)—*Colorado Medical Journal.*

---

### DISCOVERY OF "UREINE"—THE PRINCIPAL ORGANIC CONSTITUENT OF URINE, AND THE TRUE CAUSE OF UREMIA.

The following is an abstract of a paper read by Dr. Wm. Ovid Moor, of New York, during the Thirteenth International Medical Congress, at Paris, August 2–9, 1900, as we find it in the *Medical Press and Circular,* September 26, 1900. The "discovery," if further observation proves it to be such, is a very important one to urinologists, chemists and practitioners of medicine, helping greatly in the explanation of those grave manifestations and symptoms of uremia and its consequences. He says:

I recently discovered that human urine contains a liquid organic body, in a quantity superior to urea. This organic liquid is the most characteristic component part of urine. I propose to give it the name of "ureine." It is not surprising that the existence of a metabolism-product of such great importance should until the present have escaped our knowledge, for every urinary analysis has been made with the firmly-rooted idea that urine is a liquid composed of water and of inorganic solid ingredients.

In isolating this organic liquid, two principal rules should be kept in view: (1) High temperatures must be avoided. (2) Chemicals should be used as little as possible.

The urine to be examined for its liquid organic component *ureine,* should be put in a large, shallow, flat recipient and evaporated at a temperature of not over 50° C. As soon as we observe that there is no more vapor ascending from the recipient, we treat the remainder of the liquid with a strong solution of silver nitrate until no more precipitate is formed. We now cool off the liquid sufficiently to promote the separation of the saline and earthy phosphates, and then filter, washing out the filter once or twice with water, until the liquid comes out perfectly colorless. The filtrate is now put into a small cup, which should be rather deep, and heated to 65° C., in order to evaporate the remaining water. New aqueous vapor will be formed, but soon the formation of vapor will cease, though the evaporation may still continue invisibly.

To determine accurately whether or not the rest of the liquid contains a considerable percentage of water, the following delicate test is indispensable:

A large mercury thermometer is placed in the liquid and rapidly withdrawn just at 65° C.; we will probably see a puff of vapor ascending from the mercury bulb, which is an indication that the remainder of the urine still contains on undesirable quantity of water. This procedure is repeated at short intervals, and finally we shall arrive at a point when there is no more vapor ascending from the mercury bulb. We now measure the quantity of liquid which remained and add to it one-half of its volume of absolute alcohol, together with powdered pure oxalic acid, 1 gm. for each 100 c.c. of urine used, and after having allowed the newly formed oxalate of urea—$(CH_4N_2)_2C_2H_2OO_4$—to settle completely, we carefully add a concentrated alcoholic solution of oxalic acid (which is prepared by dissolving 3 gm. oxalic acid in 10 c.c. of hot absolute alcohol) until no further precipitate is formed. The alcoholic liquid thus obtained is filtered, the filter is washed out once or twice with absolute alcohol, and the whole is now exposed to a temperature of about 55° C. (but not over) for about one hour, or an hour and a half, being stirred once in a while with a horn spoon or with a glass rod. To facilitate the separation of sulphates and of the other remaining solid ingredients from the liquid organic constituent of urine—ureine—we now reduce the rest of the liquid to a low temperature and filter the same, freely using cold absolute alcohol to facilitate filtration. There is finally nothing but ureine, together with coloring matters left in the recipient (besides the alcohol, of course). To separate the coloring matters from the ureine, the alcoholic solution containing both must be treated carefully with a saturated solution of nitrate of mercury—$Hg(NO_3)_2$

—until no further precipitate is formed, and neutralized with carbonate of sodium. (It is advisable then to add a sufficient amount of sodium carbonate, so as to render the liquid slightly alkaline.) Filter again and evaporate carefully, resorting to a temperature of not over 55° C. We have thus obtained *ureine*, the liquid organic component of urine, that mysterious chemical body which is the cause of the intense blue reaction resulting from urine and a solution of red prussiate of iron and perchloride of iron.

Ureine resembles in aspect olive oil, is of a pale yellow color, of a slightly bitter taste, gives to the touch the impression of a fatty substance, and produces on paper spots resembling fat spots, though not so marked as those produced by fat. Its specific gravity is about 1270; it is, therefore, much heavier than water and about as heavy as glycerine. It is freely miscible in all proportions with water and alcohol, whether these latter are of a neutral, acid, or alkaline reaction; it is barely soluble in ether. Its own reaction is very slightly alkaline, almost neutral.

My preliminary investigations have led me to the conclusion that this organic metabolism-product of the human body belongs to the group of alcohols of the aromatic series; at a temperature of about 80° C. it begins to split into several bodies belonging to the class of aromatic oxy-acids, and if heated to about 150° C. it leaves behind pure carbon. This organic liquid has a characteristic odor; in fact, it is this constituent of urine which is the cause of its specific odor. Rubbed into the skin it soon produces a sensation of slight burning; there can be no doubt, therefore, that it is the principal cause of the irritating qualities of urine. One of the most remarkable characteristics of ureine is its ability to take up large quantities of oxygen with great facility. The quantity of 50 c.c. of the average human urine can deoxidize 1 gm. of potassium permanganate, and one sample of urine taken from a woman in the ninth month of pregnancy deoxidized with great rapidity over 4 gm. of permanganate to each 100 c.c. of urine; this urine was voided about five o'clock in the afternoon, was of an intense yellow color and of a specific gravity of 1030; it contained about 2¾ per cent. (by volume) of ureine. It is important to remember that the power of urine to take up oxygen does not depend only on the quantity of ureine present, but to a greater extent on the quality of the ureine, on some intrinsic peculiar force inherent in this wonderful organic liquid; I have seen urine, containing less ureine than did other samples of urine, absorb more oxygen than did the latter samples which were richer in ureine, and especially strong was this power of absorption in the urine of a pregnant woman. Urine which has been subjected for some length of time to a temperature of 70°--80° C. loses in a great measure its capacity of taking up oxygen, and when exposed to 90° C. for about half an hour it loses 75 per cent. of its absorptive power. It does not take up at once all the oxygen which it is able to absorb, but does so with great avidity in the beginning, and gradually takes up less and less during equal periods of time; its capacity of absorbing oxygen is not wholly extinguished before a lapse of four or five weeks. Ureine is present in a quantity double that of urea; the greatest amount of ureine is contained in urine voided between 5 and 7 p. m.

It was to be expected *à priori* that the principal organic constituent of urine should be the true cause of those complex toxic symptoms which have been designated by the collective name of uremia, and which were quite frequently incidental to the puerperal state, after all the other organic and inorganic components of urine failed to account for these terrible toxic phenomena. A few experiments on rabbits have fully demonstrated the truth of this *à priori* conclusion. Several of these animals, each weighing over 1 kgm., have succumbed after three to eight hours from subcutaneous injections of 3½ to 4½ c.c. of ureine.

Ureine is, I believe, the principal cause of the ammoniacal fermentation of urine, as without its presence urea cannot be decomposed into ammonia and carbon dioxide. Neither Pasteur's micrococcus ureæ, nor Leube's bacterium ureæ, nor any other micro-organism is able to change urea; in fact, in many respects urea is just as indestructible as iron, silver, or any other element, for the strongest mineral acids do not decompose it, but simply combine with it. Only a temperature of above 130° C., perhaps 140° C., can split urea into ammonia and carbon dioxide. Ureine is, therefore, a ferment, which has a potential energy of at least 130° C. Without ureine all organic matter would become converted into urea, which would remain in nature without any use, and thus within a limited period of time all vegetation and animal as well as human life would cease.— *Virginia Medical Semi-Monthly.*

---

### CEREBRO-SPINAL FEVER.*

Cerebro-spinal fever, or cerebro-spinal meningitis, as a distinct entity, is much less prevalent than most of the contagious or infectious diseases, yet it may be considered to be endemic in the United States, for no year now passes without a greater or less number of deaths from this cause being reported, especially in the larger cities. I trust, therefore, that a review of the subject may be of some interest.

Cerebro-spinal fever did not exist, or was not recognized, until the beginning of the nineteenth century, during the first half of which there is record of various epidemics, both in this country and in Europe. At about 1860 it had become established in America so that each year brought reports of the disease in some of our cities, and it would seem that the number of cases has increased more rapidly in proportion than has the population. In 1872 a severe epidemic occurred in New York city, a year or two later it visited this part of the State, and New England in 1893. It is impossible to say to just what extent the malady is prevalent in this locality, as cases of it are not reported to the health authorities, yet in the city of Rochester for the last four years an annual average of eighty-eight deaths due to "meningitis," as indifferently classified, is recorded.

Nothing has been adduced in recent years to revise the belief that it is practically a non-contagious disease. A few cases are recorded in the literature of the subject where the disease seemed to have been communicated from one person to another, but the common history even in epidemics is that of only one member of a family being attacked, while on the other hand, it may be breaking out in households separated by long distances and holding no communication whatever with each other. J. L. Smith states that in one case only in his own practice did the facts point to contagious-

*Read by F. B. Maynard, M.D., Rochester, N.Y., at the annual meeting of the Monroe County Medical Society, held at Rochester, N.Y., May 29, 1900.

ness. He further says: "An interesting fact in regard to these many epidemics on both continents is that they have occurred in isolated localities, far apart and without the least evidence of transportation. Cerebro-spinal fever resembles in this respect the diseases due to marsh miasm."

Monk (*British Medical Journal*, July 30, 1892) reports an epidemic which affected children only. The origin was traced to a village school where a number of the children had been affected by diarrhea a few weeks previously. Disinfection and closing of the school temporarily caused a cessation of the epidemic.

Meriwether (*North Carolina Medical Journal*, July 7, 1888) reports an epidemic of meningitis that prevailed in North Carolina during the early part of the year 1888. He says that, "not one of ninety-nine cases of which notes were taken, lived on high well-drained ground. Nearly all the cases occurred in families using well water." In only ten per cent. of the cases could the sanitary condition of the dwellings be classed as good.

Class (*Medical News*, December, 1898) puts cerebro-spinal meningitis in the same category with phthisis pulmonalis, and says that, "unsanitary conditions afford the proper nidus for the growth of the germ of this disease."

While, therefore, isolation of the patient is quite unnecessary, yet it seems to me advisable that this disease be added to the list of those to be reported to local boards of health in order that we may be kept informed of the degree of its prevalence, and that we may know more of its geographical distribution in the city or town. In this way the attention of the health authorities would be called to its predominance in a certain locality, if such a condition should become apparent, and a more careful inquiry into the conditions there causing it or favoring its propagation would result. We might thus gain some valuable information as to its etiology, and something might be done toward its prevention.

This disease occurs oftener among children than among adults. J. L. Smith states that three-fourths of the cases in his practice were under ten years of age. The majority of cases occur during the winter and spring months.

In 1887 Weichselbaum described a diplococcus which he had found in the meningeal pus of six cases of acute cerebro-spinal fever, but he was unable to reproduce the disease in animals. Jaeger, in 1895, obtained similar results from his investigations.

Councilman has studied the disease carefully during recent years in New England, and in 1898 (*Medical Record*, April, 1898, and *Report to State Board of Health*, 1898) described a micrococcus, very like the gonococcus in appearance, which now seems to be the specific cause of the disease, although Connor and others have failed to find it in every case examined, and in others have found it in conjunction with the pneumococcus. Councilman describes it as an organism—designated as the "diplococcus intracellularis"—of about the size of the ordinary pathogenic micrococci, appearing in diplococcus form as two hemispheres separated by an unstained interval. It takes the ordinary stains but is decolorized by Gram's method, and is thus differentiated from the diplococcus lanceolatus of Fraenkel.

Kiefer had already, in 1896 (*Berliner klinische Wochenschrift*, November 28, 1896), described the same organism and stated that although it was impossible to produce cerebro-spinal meningitis in guinea-pigs by inoculation, yet while carrying on the observations "the operator had rhinitis with marked depression, headache, nervousness and a drawing pain in the neck. The pus from the nose showed cocci similar to those in the cultures and these soon overcame the other bacteria in the nose, appearing in almost pure culture."

Sears (*Boston Medical and Surgical Journal*, August 8, 1888) called attention to the frequency of pharyngitis during epidemics of cerebro-spinal meningitis, and Wolf (*Berliner klinische Wochenschrift*, March 8, 1897) believes that one of the most frequent modes of infection is from the micro-organism's gaining access through the naso-pharynx to the Eustachian tube, to the middle ear and thence into the cranial cavity.

The exact path of infection is still problematical but the nasal cavity seems to be the most suspected portal.

Stillé aptly called cerebro-spinal meningitis a chameleon-like disease. Its onset may be gradual, although it is usually sudden, and, it has been observed, rarely begins in the early morning after a quiet night's sleep, beginning, rather, in the afternoon or evening hours. Commonly it is ushered in with vomiting, headache and rigor. Instead of the chill, convulsions, more or less severe, followed by stupor, may occur in children. Neuralgic pains, shifting from one part to another, supervene. Among early symptoms are those in which the eye is concerned—dilatation, or inequality, or, more rarely, contraction of the pupils, with reaction to light sluggish or absent. Nystagmus is sometimes present; photophobia is almost constant, as is also strabismus.

Vomiting—frequently an initial sign—is usually reported to be projectile in character, but Connor (a paper read before the New York Neurological Society, January 3, 1889) says that in sixty cases observed in New York hospitals the vomiting was in no case projectile; that it was present at some time in over half the cases. Constipation is very frequently present from the onset. The tongue is usually but slightly furred.

As the disease fastens itself upon the patient the face takes on a fixed expression, indicative of intense suffering. There are spasmodic contractions or convulsive movements of the muscles of the extremities. As the disease progresses the condition of the patient's mind may go through every grade of disturbance from coma to delirium—now he may lie apathetic and indifferent to surroundings, and shortly rouse up and begin a persistent rolling of the head from side to side, moaning piteously and tossing about the bed.

The patient becomes greatly and quite rapidly emaciated. Hyperesthesia of the surface is a common and important sign, even a light touch eliciting a moaning cry of pain. In no disease with which cerebro-spinal meningitis is apt to be confounded is this symptom so prominent and constant. Muscular contraction is not an initial symptom, but in typical cases is constant after the second or third day, when we find the patient with head retracted, the back more or less arched and extremities flexed. Kernig has called attention to a sign that is of a good deal of diagnostic value, viz., that with the thigh semiflexed the leg cannot be extended upon the thigh. The abdomen is often retracted, but this is not a constant sign. Paralysis occasionally occurs in one or another set of muscles.

We often see herpes labialis, and the *tache cérébrale* is a common but not important sign, as it may occur in other conditions.

In Connor's sixty cases skin manifestations of one kind or another were observed in twenty-five per cent., but hemorrhagic eruption was seen in only five per cent. This latter manifestation, from which the name "spotted fever" was derived, has apparently been much less frequently seen since the epidemic of 1872 than previously.

The temperature runs differently in different cases. Usually there is considerable surface heat, especially of the head, and a fairly high body temperature. In some cases the temperature runs normal or subnormal; in many it fluctuates widely and may be highest in the morning. Pyrexia may subside some days before the nervous symptoms abate.

The pulse varies greatly, although retardation is not common. In typical cases in children we may expect the pulse to be between 130 and 150, and respirations about forty per minute.

The amount and quality of the urine are not far from normal, but we should be on our guard against retention.

Of the special senses sight and hearing are the ones apt to be affected, and one or both of these are not infrequently irretrievably lost. These are the most probable and serious sequelæ to be expected.

The mortality ranges, according to various observers, from thirty per cent. to eighty per cent.; with children the death-rate is much higher than in adults. It has been well said that there is probably no disease which falsifies the predictions of the physician more frequently than cerebro-spinal fever.

In the treatment of a case we have the proposition before us of a constitutional disease, asthenic and protracted in nature, with characteristic involvement of the meninges of the brain and cord, and a profound disturbance of the whole nervous system. On account of the experiences recorded where blood depletion has been practiced, even early in the disease, it is apparent that the hemogenetic function of the body is in large measure suspended, so that while the use of leeches may temporarily relieve the congestion of the nerve centers the patient loses vital fluid that is not replaced, and will probably die of sheer exhaustion before the disease shall have run its course. Depletion is, therefore, to be condemned.

As we are coming to regard more the systemic invasion in these diseases of microbic origin—each characterized by its local lesions—our system of treatment has broadened somewhat. Little has been said in advocacy of the use of cold affusions or the cold pack applied to the whole body. Ice to the head and hot water to the rest of the body has seemed, in view of the cerebral congestion, to be the only rational hydrotherapy. But the remarkably soothing and tonic effect on the nervous system, especially in children, and the increased elimination from the body of the organic poisons following the use of the cold bath in typhoid and other fevers, suggests, by analogy, its use in the disease under consideration. In a case that has been recently under my care—a child four and a half years of age—the cold pack was used with most satisfactory results. One night in the early stage of his sickness I found the boy tossing about the bed and moaning in a most distracting fashion—muscles of the extremities contracted and the face drawn down and expressive of great suffering; axillary temperature 104°, pulse rapid and not strong. I had him stripped and placed in a twice folded sheet wet in water as it ran from the faucet, with a rubber bag two-thirds full of water and crushed ice, replaced by another as it grew warm for a pillow. In less than five minutes the child had subsided into a quiet, natural sleep, which continued for over two hours. This was repeated at intervals depending upon the temperature and nervous condition until the fever abated, and I firmly believe that it did much to carry the child through the most critical stage of the disease and to his ultimate recovery. It would seem, however, that the length of time to continue the use of the cold pack and the occasion for using it require greater discrimination in this than in the other fevers.

Strumpell recognizes the availability of cold baths, and Barr (*British Medical Journal*, November 18, 1899), recommends the ice cap of Leiter's tubing, and in case this is not enough to reduce temperature applies cold compresses to the abdomen or runs a small stream of tepid water over the body, the cold to be continued to the head until the temperature is for some time subnormal. J. C. Wilson admits the use of "gentle cold affusions"; this is also recommended by German writers. Osler advocates cold baths when the temperature goes above one hundred and two and one-half degrees, reasoning from the value of hydrotherapy in typhoid fever.

The constipation yields readily to calomel in small, repeated doses.

Potassium bromide is a drug used by J. L. Smith, through a long experience, with great satisfaction. Children, especially, take it well, and there is little danger of bromism as it is rapidly eliminated. Mary Putnam-Jacobi has shown that it causes contraction of the small vessels of the nerve centers and diminishes hyperemia; it also diminishes in a marked degree the reflex irritability of the cord and is consequently of great service in combating the extreme nervous excitation. In my case, when the temperature of the patient did not indicate the use of the cold pack, bromide of potassium proved most useful. At one time it was being administered in doses of five grains every two hours.

Iodide of potassium is probably of some value in hastening absorption of the exudate after the acute stage of the disease has passed. The use of morphine is widely and favorably regarded. In the case referred to I administered it on only two occasions, in one-sixteenth grain doses. Alcoholic stimulation is usually well borne and should be used when indicated.

McNabb (*New York Medical Journal*, February 25, 1899), reports on the use of antistreptococcus serum in a few cases, and believes that among other points in its favor it prevents purulent infection.

Lumbar puncture has its advocates, but is, after all, of diagnostic value only.

Schirmer (*New Yorker medicinische Monatsschrift*, November, 1898) and Dixon (*Journal of the American Medical Association*, May 27, 1899) report very favorably on the use of Credé's ointment by inunction. It has been shown that the soluble silver is absorbed through the skin, and that in the presence of pyogenic germs and their toxins it enters into some combination and acts as a germicide or antitoxin. Credé himself (*Klinische therapeutische Wochenschrift*, 1898) says that with children, who absorb it more quickly than adults, twenty to twenty-five minutes of constant friction is required to insure its absorption. This is certainly an objectionable feature of its use in view of the intense hyperesthesia that attends the disease. But the treatment promises something and is worthy of further investigation.

In the treatment of cerebro-spinal fever, as in so many other diseases, various remedial agents are lauded, but until the value of something new has been more clearly demonstrated, good nursing, easily assimilable nutriment in plenty, a quiet and darkened room for the patient, intelligent hydrotherapy, and the bromide of potassium will be, it seems to me, our mainstays.

---

### Cyclic Albuminuria of Adolescents.

Dr. H. Sanchez (*International Congress of Medical Sciences*) reported two cases of this disorder and showed that the affection may attach itself at times to a diathesis, particularly gout, and sometimes to an infectious cause, such as typhoid or tonsillitis. The first variety is distinguished from nephritis by its systematic periodicity, the absence of general troubles and of urinary casts, freedom from uremia, and perfect cure without special régime. The second variety is often preceded by hematuria, then albuminuria, which may be cyclic or constant. Arthritism, gout, the menstrual flow and adolescence predispose to this renal fluxion, without fever. The pyrexias recall it, and pregnancy may bring it forth. Without very strict and prolonged hygiene, absolutely necessary for a cure, marriage ought not to be counseled. Dr. Sanchez thinks that cyclic albuminuria is very often overlooked, being only functional trouble at the outset, and when recognized it is transformed into nephritis with uricemia. In every instance this variety of cyclic albuminuria ought to be considered as a type by itself, and it should not be termed intermittent albuminuria, since this is a character of all common albuminurias capable of cure.—*Denver Med. Times.*

